# IART^®^ (Intra-Operative Avidination for Radionuclide Therapy) for accelerated radiotherapy in breast cancer patients. Technical aspects and preliminary results of a phase II study with 90Y-labelled biotin

**DOI:** 10.3332/ecancer.2010.166

**Published:** 2010-11-01

**Authors:** G Paganelli, C De Cicco, M E Ferrari, G McVie, G Pagani, M C Leonardi, M Cremonesi, A Ferrari, M Pacifici, A Di Dia, F Botta, R De Santis, V Galimberti, A Luini, R Orecchia, U Veronesi

**Affiliations:** 1 Division of Nuclear Medicine; 2 Division of Medical Physics; 3 Scientific Directorate; 4 Division of Senology; 5 Division of Radiotherapy, European Institute of Oncology, via Ripamonti 435, Milan, Italy; 6 Sigma-Tau SpA R&D, Via Pontina Km 30.400, Pomezia, Rome, Italy; 7 University of Milan, School of Medicine, Milan, Italy

## Abstract

**Background:**

Breast conserving surgery (BCS) plus external beam radiotherapy (EBRT) is considered the standard treatment for early breast cancer. We have investigated the possibility of irradiating the residual gland, using an innovative nuclear medicine approach named IART^®^ (Intra-operative Avidination for Radionuclide Therapy).

**Aim:**

The objective of this study was to determine the optimal dose of avidin with a fixed activity (3.7 GBq) of ^90^Y-biotin, in order to provide a boost of 20 Gy, followed by EBRT to the whole breast (WB) at the reduced dose of 40 Gy. Local and systemic toxicity, patient’s quality of life, including the cosmetic results after the combined treatment with IART^®^ and EBRT, were assessed.

**Methods:**

After tumour excision, the surgeon injected native avidin diluted in 30 ml of saline solution into and around the tumour bed (see video). Patients received one of three avidin dose levels: 50 mg (10 pts), 100 mg (15 pts) and 150 mg (10 pts). Between 12 to 24 h after surgery, 3.7 GBq ^90^Y-biotin spiked with 185 MBq ^111^In-biotin was administered intravenously (i.v.). Whole body scans and SPECT images were performed up to 30 h post-injection for dosimetric purposes. WB-EBRT was administered four weeks after the IART^®^ boost. Local toxicity and quality of life were evaluated.

**Results:**

Thirty-five patients were evaluated. No side effects were observed after avidin administration and ^90^Y-biotin infusion. An avidin dose level of 100 mg resulted the most appropriate in order to deliver the required radiation dose (19.5 ± 4.0 Gy) to the surgical bed. At the end of IART^®^, no local toxicity occurred and the overall cosmetic result was good. The tolerance to the reduced EBRT was also good. The highest grade of transient local toxicity was G3, which occurred in 3/32 pts following the completion of WB-EBRT. The combination of IART^®^+EBRT was well accepted by the patients, without any changes to their quality of life.

**Conclusions:**

These preliminary results support the hypothesis that IART^®^ may represent a valid approach to accelerated WB irradiation after BCS. We hope that this nuclear medicine technique will contribute to a better management of breast cancer patients.

## Introduction

Breast conserving surgery (BCS) to excise the tumour with adequate margins followed by whole-breast external beam radiotherapy (WB-EBRT) is now considered the standard treatment for the majority of patients with early breast cancer [1–3].

The post-operative radiotherapy schedule that follows BCS is usually delivered in daily fractions each weekday for a period of six to eight weeks. This can represent a logistic problem for many patients, particularly the elderly and those who reside a considerable distance from a radiation treatment facility [4, 5].

The radiotherapy schedule currently recommended consists of 45–50 Gy at 1.8–2 Gy per fraction to the WB, followed by a 10–16 Gy boost to the tumour bed [6].

Based on the results of several prospective randomised clinical trials comparing BCS with or without WB-EBRT, locoregional recurrence occurs in approximately 10%–35% of women receiving BCS alone and in 0.3%–8% of women after BCS plus WB-EBRT (follow-up range: 39–102 months) [2, 3]. Although the WB-EBRT impact on survival is uncertain, for most patients who choose BSC instead of mastectomy, reducing the risk of local recurrences matches the therapeutic priorities and preferences that motivated their choice of BCS. Nevertheless, evidence suggests that up to 30% of patients who undergo BCS for early breast cancer did not receive, for some reason, post-operative breast irradiation.

As an alternative to conventional WB-EBRT, partial breast irradiation (PBI) using intra-operative radiotherapy (IORT) or other modalities such as MammoSite, brachytherapy, high-conformal EBRT have been recently proposed [7–11] and are currently under clinical evaluation. As yet, there is no firmly established standardised IORT dose for PBI in early breast cancer. Although valid, PBI presents two major limitations: (a) the availability of dedicated devices such as an intra-operative linear accelerator, MammoSite balloon, etc. and (b) a restricted field of irradiation, which limits the management of positive surgical margins and unsuspected clinical neoplastic foci far from the primary tumour.

Previous experiences in locoregional treatment of peritoneal carcinomatosis and malignant gliomas, using ^90^Y-Biotin with an avidin-based pre-targeting technique [12–14], lead us to assume that the application of a radionuclide therapy, with radio-labelled biotin, might also represent an alternative approach to deliver radiation (beta and alpha particles) to the operated breast. We thus developed a new technique named IART^®^: *Intra-operative Avidination for Radionuclide Therapy* that relies on the avidin–biotin binding system.

Avidin is a 66 kDa highly glycosylated and positively charged (isoelectric point, pI ≅ 10) tetrameric protein, which is extracted from the white of the egg, which shows extremely high affinity for the 244 Da vitamin, biotin (*k_d_* = 10^−15^ M).

Briefly, the ‘avidination’ of the operated breast with native avidin, directly injected by the surgeon, into and around the tumour bed, provides a target for the radio-labelled biotin intravenously [15] injected one day later.

Furthermore, it is well known that the inflammatory reaction displays cation-exchanging properties [16–18]. Thus, at the surgical site, avidin is retained for several days, acting as an ‘*interstitial molecular device*’ able to bind radio-labelled biotin. The proof of the principle of this new approach was documented in a biodistribution and pharmacokinetic studies conducted in ten patients who underwent BCS. The IART^®^ dosimetry with ^111^In-Dota-biotin according to the Medical Internal Radiation Dose MIRD formula and the linear quadratic model (biological effective dose, BED) was previously reported [15, 19, 20].

We present here the results of a phase I–II study in which IART^®^ was applied to deliver a 20 Gy dose with ^90^Y-labelled biotin, as an anticipated boost to a shortened EBRT course, in women who underwent BCS for breast cancer.

Primary objectives of this study were:

- to determine the optimal dose of avidin with a fixed activity (3.7 GBq) of ^90^Y-ST2210 in order to provide a dose of 20 Gy;
- to assess local and systemic toxicity and to evaluate the pharmacokinetics of different amount of avidin.

A secondary objective was to assess patient’s quality of life (QoL), including the cosmetic results, after the combined treatment IART^®^ and reduced EBRT.

## Material and methods

Thirty-five post-menopausal women with breast cancer suitable for BCS were enrolled. Inclusion criteria were: age ≤ 75 years; diagnosis of breast cancer on the basis of cytology, histology and mammography/echography; tumour size ≤ 5cm with clinically negative axillary lymph nodes and absence of distant metastases (T1–2, cN0, M0); Karnofsky performance status ≥ 70%; adequate haematological function: WBC ≥ 3000/mm^3^, ANC ≥ 2500/mm^3^, Hb ≥ 10 g/dl; platelets ≥ 150,000/mm^3^; serum creatinine ≤ 1.5 × UNL; normal liver function: total bilirubin ≤ 1.5 × UNL; alkaline phosphatase ≤ 2.5 × UNL and ALAT and/or ASAT ≤ 1.5 × UNL.

Patients with non-infiltrating tumour of the breast, Paget’s carcinoma or a non-carcinoma histotype were excluded from the protocol. Other exclusion criteria were previous excisional biopsy on the same breast, history of neoplasia, insulin-dependent diabetes mellitus, hypertension not pharmacologically controlled, previous treatment with avidin, referred allergy to eggs, poor compliance with radiotherapy and the follow-up program, concomitant treatment with experimental drugs.

This study was conducted according to the ethical principles, which are derived from the Declaration of Helsinki, from the Good Clinical Practice guidelines and from applicable legislation. The protocol was started after Ethical Committee approval (Study Protocol IEO S313/406).

Before entering the study, each candidate accepted to participate in the study by signing the informed consent form.

From March 2007 to March 2008, 38 post-menopausal women were selected. Thirty-five patients were enrolled. Three patients were excluded from the study due to inadequate haematological function (platelets <150,000/mm^3^), abnormal liver function (ALAT and ASAT >1.5 x UNL) or the presence of a non-infiltrating carcinoma. The mean age of the treated patients was 63 years (range 42–74).

All patients underwent partial breast resection (quadrantectomy). Sentinel node biopsy alone was carried out in 28 patients; 7 patients had sentinel node positive and axillary dissection followed. Pathological examination demonstrated an infiltrating ductal carcinoma in 24 cases, 2 mucinous carcinoma, 3 infiltrating lobular carcinoma, and an infiltrating tubular carcinoma in 1 case, 2 infiltrating mixed carcinoma, 1 infiltrating papillary carcinoma; in two patients an intra-epithelial neoplasia was diagnosed as DIN1 c, DIN G2, respectively. One patient underwent breast reduction for cosmetic purposes after tumour removal. All patients received avidin administration in the surgical bed as previously described [15, 20]. Briefly, after the primary tumour excision, the surgeon injected native avidin diluted into 30 ml of saline solution into and around the tumour bed, by means of three syringes (10 ml each). Two syringes were used for intra-parenchyma injection (every 1.5 cm) and the third for the resection margins, after having closed the gland.

Three consecutive cohorts of patients were treated according to the protocol (Table 1).

The starting dose of avidin (100 mg) was based on the results of previous studies [15, 20]. In the present study, the lower avidin dose (50 mg) and the higher avidin dose (150 mg) were also investigated in order to identify the optimal amount able to provide a 20 Gy dose to the tumour area, after a fixed activity of ^90^Y-biotin (3.7 GBq).

After avidin administration (mean time 18 ± 3 h) and before the i.v. injection of radioactive biotin, 5,10 or 15 mg (1/10 of the avidin dose) of biotinylated human serum albumin (HSA-biot) were administered intravenously for 5 min. This biotinylated chase was intended to reduce circulating avidin as previously described [20]. Thereafter, 3.7 GBq of ^90^Y-ST2210 (specific activity of 4 GBq/mg) together with 185 MBq of ^111^In-biotin (specific activity of 4 GBq/mg) was delivered intravenously by slow infusion, using a dedicated disposable system [21].

**Figure:**
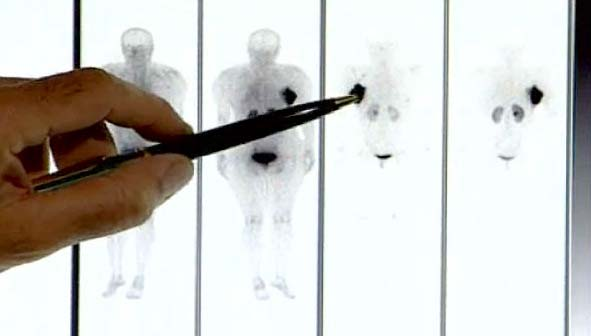
To view this video click here: http://www.ecancermedicalscience.com/view-article.asp?doi=10.3332/ecancer.2010.166

## Imaging, biodistribution and dosimetry

Total body scintigraphic images were collected after 1.5 ± 0.5, 5 ± 2, 16 ± 4 and 24 ± 6 h after the administration of the radio-labelled biotin, using a double-head γ-camera (GE, Infinia II) equipped with a medium-energy general-purpose collimator (MEGP). A SPECT image was acquired after 16 ± 4 h in order to provide maps of activity distribution of the index quadrant. A low-dose CT was also acquired in order to evaluate the patient-specific organ masses, especially the kidneys.

**Figure: 1:**
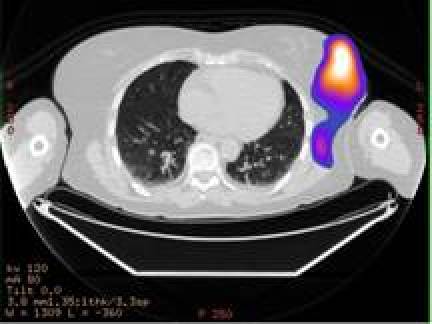

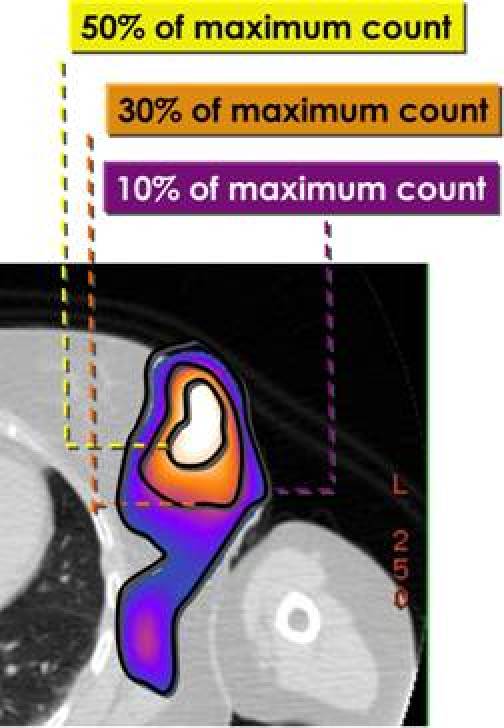
a: SPECT/CT fused images showing a hot region in the operated left breast. b: The hot region was divided into three different areas: high-uptake area (uptake higher than 50% of the maximum—isorois 50%); medium-uptake area (between 50% and 30% isorois) and low-uptake area (between 30% and 10% isorois).

In the diagnostic phase [20], the attention was focused on biodistribution, pharmacokinetics and dosimetry in normal organs. In this phase I–II study (Study Protocol IEO S313/406), we focused on dosimetry of the breast region and of the critical organs (kidneys). For this purpose, functional images (SPECT) have been fused to morphological images (CT), using the workstation Advantage Windows (GE Medical System). Before SPECT, three radioactive and radio-opaque markers were positioned on the skin of the patient to allow appropriate image matching. SPECT/CT fused images were used to assess the correct tracer localisation in the breast (Figure 1a).

Dosimetry was performed as previously described [15]. Time activity curves were obtained for the normal organs (whole body images) and the breast gland (SPECT and whole body images). In particular, the breast region was divided into three different areas (Figure 1b).

- High-uptake area (uptake higher than 50% of the maximum—isorois 50%);
- medium-uptake area (between 50% and 30% isorois);
- low-uptake area (between 30% and 10% isorois).

For each patient, dose calculations were performed entering the number of decays (ND) estimated for normal organs (kidneys, heart, lungs, red marrow, urinary bladder contents and remainder of the body) and irradiated breast in the OLINDA/EXM software [22], applying the correction for the individual patient weight and organ masses. In particular, the self-dose in the three breast areas were calculated considering the ND values derived for the three areas identified (high, medium, low uptake), with the approximation of uniform activity distribution in lesions (OLINDA/EXM, sphere model).

Finally, to analyse the differences between the three consecutive cohorts of patients, the following parameters related to the breast area were compared:

- maximum uptake (%) of injected activity in the breast area;
- number of decays in the breast area showing high uptake;
- absorbed dose per unit activity in the breast area showing high uptake.

## Biological effective dose: the linear quadratic model

The linear quadratic model enabled the comparison of doses released through EBRT to those from IART^®^ for the analysis of possible effects on the operated breast and non-target organs. In particular, we adopted the BED expression defined by [19]:

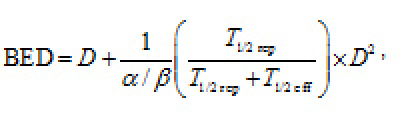
where *D* is the dose delivered, *T*_1/2 rep_ is the repair half-time of sublethal damage and *T*_1/2eff_ is the effective half-life of the radiopharmaceutical in the specific tissue. The α/β ratio relates the intrinsic radiosensitivity (α) and the potential sparing capacity (β) for a specified tissue or effect. For the breast region, we used a *T*_1/2 rep_ equal to 1.5 h, α/β 10 Gy; for the kidneys, we used a *T*_1/2 rep_ equal to 2.8 h, α/β 2.6 Gy [20, 23, 24].

## Radiation therapy

Post-operative external-beam radiation was delivered to the entire breast four weeks after IART^®^. Patients were treated in the supine position, placed on a breast board, with both arms raised above head. A 6-MV photon intensity was used to deliver a dose of 40 Gy: 2.0 Gy, fractions for five days/week for four weeks, with an isocentric technique. The target volume was the entire breast parenchyma, extending from 5 mm below the skin surface to the deep fascia, excluding the underlying muscle and rib cage. The tangential technique using a parallel pair of radiation beams angled anteriorly to produce coplanar posterior field edges was employed. The breast was included in the 95% isodose line and the dose maximum was documented on the treatment plan. Wedges and appropriate shielding were used and dose-volume histograms for organs at risk were plotted.

## Results

**Figure 2:**
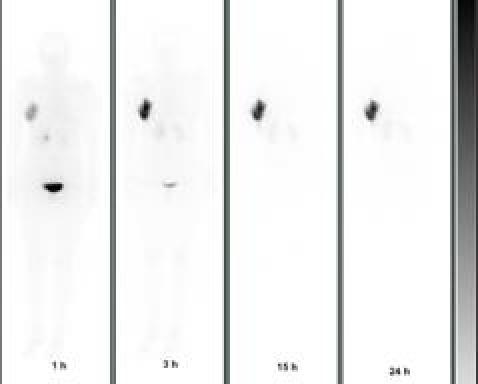
Whole body scans (anterior projection) acquired at 1, 3, 15 and 24 h post-injection of ^90^Y-biotin in a patient operated in the right breast.

The WBS images (Figure 2) showed a fast and intense uptake of the radio-labelled biotin in the breast area of all patients. Dosimetric data and estimated masses of the irradiated breast, obtained for the three cohorts of patients, showed that a considerable mass of breast parenchyma, with a mean value of 250 g, was irradiated using 100 or 150 mg of avidin. Data from the 15 women, given 100 mg of avidin, showed a mean absorbed dose to the breast of 19.5±4.0 Gy in the area of highest uptake, with a corresponding BED of 21.2±4.3 Gy. The dose of 100 mg of avidin resulted to be the most appropriate (and the minimum needed) in terms of dose delivery into the index quadrant. The gap between surgery and biotin injection did not influence the percentage uptake in the operated breast, which reached 12% with a mean value of ∼ 8% (see Table 2).

The absorbed dose to the kidneys after injection of 3.7 GBq of ^90^Y-DOTA-biotin was 3.8 ± 1.1 Gy (1.0 ± 0.3 Gy/GBq), comparable with the results obtained in the previous study (1.2 ± 0.4 Gy/GBq) [15]. Moreover, the estimated BED for the kidneys was 4.3+1.7 Gy, well below the 44 Gy reported in the literature as the TD_50/5_ for renal damage [24]. The absorbed dose to the red marrow was 0.2 ± 0.1 Gy, never exceeding 0.4 Gy, giving no concern for haematological toxicity.

## Toxicity and quality of life

No side effects were observed after avidin administration and biotinylated HSA chase, nor after radio-labelled biotin, either ^111^In or ^90^Y labelled. Systemic toxicity, assessed by evaluating haematological, liver and renal functions, was not found. The overall cosmetic result was good.

**Figure 3:**
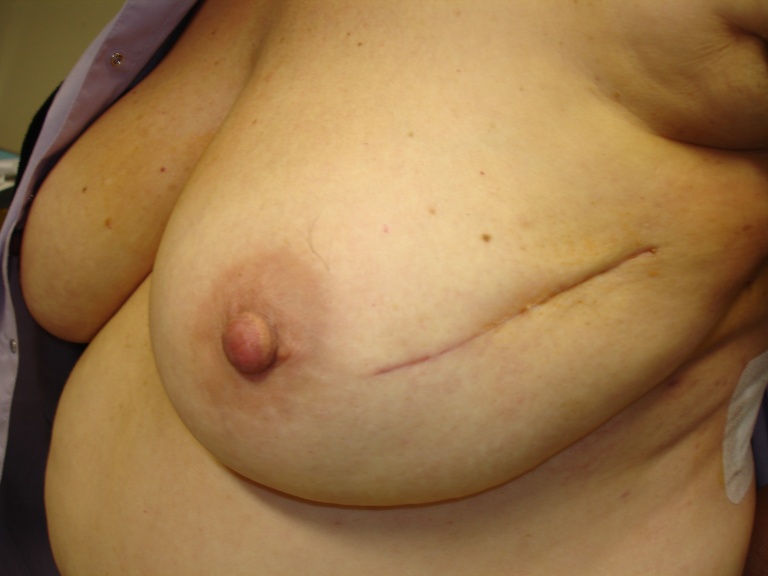
Good cosmetic results and normal wound healing in a patient who received IART^®^ alone.

Thirty-five patients received IART^®^ and 32 completed EBRT, starting four weeks after IART^®^. In one case, due to the presence of sieroma on the tumour bed, EBTR started one week later. In one patient, the course of EBRT was extended through two months instead of one month: the treatment was stopped for four weeks due to inflammation and then restarted when the process had resolved. One patient, who underwent cosmetic bilateral breast plastic reconstructive surgery, developed a bilateral delay to wound healing and so WB-EBRT commenced two months after IART^®^. Three patients did not receive EBRT: one patient underwent nipple-sparing mastectomy because of positive margins of resection after quadrantectomy; in two patients the definitive histological examination showed low-grade intra-epithelial neoplasia. The cosmetic result and wound healing of one of these patients is shown in Figure 3.

**Figure 4:**
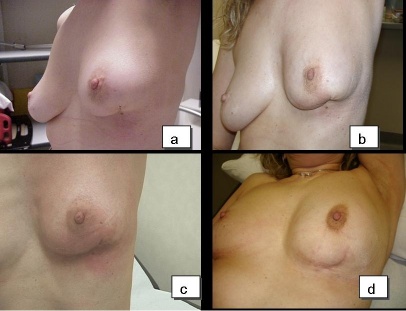
Patient who received IART^®^ plus EBRT. a: No local toxicity was observed four weeks after IART^®^; b: mild local toxicity after delivery of 20 Gy by EBRT; c: local toxicity (classified as G1) month after completion of EBRT; d: no local toxicity present six months after EBRT. Cosmetic outcome was judged good.

The compliance to EBRT and local toxicity during treatment at the one- and six-month follow-ups were assessed by evaluating local signs according to the Radiation Therapy Oncology Group (RTOG) Scale. The presence of symptoms (pain, itch and burning) was reported at each clinical control. Patients were controlled at the weekly EBRT sessions. All the 32 patients reported mild pain or/and itch in correspondence to the surgical bed during and at the end of the treatment. These symptoms resolved within one week after the end of EBRT. Table 3 reports the local toxicity results in the 32 patients who received EBRT after IART^®^. The majority of patients experienced low-grade toxicity during EBRT, starting from the second week. At the end of EBRT, local toxicity was classified as G2 in 25 patients and as G1 in 4 patients. Grade 3 skin toxicity was observed in three patients. Four weeks after the completion of WB-EBRT, no local toxicity (G0) was observed in 20 cases, while 10 patients had residual G1 and 2 patients had G2 toxicity. All patients completed the six-month follow-up and showed an excellent tolerance to the whole treatment schedule. Figure 4 depicts an example of mild local toxicity through the time, with a complete recovery six months after EBRT treatment.

Six patients underwent chemotherapy: three patients received an anthracycline and CMF scheme (cyclophosphamide, methotrexate and fluorouracil), one patient received CMF alone, one patient received vinorelbine plus Trastuzumab and the last one anthracycline plus Trastuzumab and letrozole. All patients started chemotherapy at the completion of the EBRT. The remaining 29 patients had positive hormonal receptor status and were given endocrine therapy. No significantly delayed toxicity was observed in the six subjects who received chemotherapy.

IART^®^ plus short four-week EBRT treatment was very well accepted by the patients. The quality of life, evaluated by the EORTC QoL questionnaire, demonstrated no significant changes in patients’ quality of life (Tables 4 and 5).

## Discussion

The results of this pilot study suggest that the IART^®^ is a safe nuclear medicine procedure able to deliver, one day after surgery, a 20-Gy radiation dose to women who underwent BCS for breast cancer.

The minimum quantity of avidin that reliably provides the 20-Gy target dose is 100 mg; 50 mg provides a slightly lower dose, while 150 mg does not appreciably increase it. The dose of 100 mg of avidin acts as an ‘*interstitial molecular device’* that specifically binds ^90^Y-labelled-DOTA-biotin in a considerable mass of breast tissue, without any significant side effects. IART^®^ takes advantage of the high affinity between avidin and biotin and mimics the well-known model of radiometabolic treatment with ^131^I in differentiated thyroid carcinoma.

The scintigraphic images demonstrated a fast and stable uptake of labelled ^90^Y-DOTA-biotin at the operated breast site. Moreover, in 20 patients, the axillary lymph nodes or the internal mammary chain nodes were visualised. This observation indicates that avidin, as albumin colloids for the sentinel node biopsy, is drained into the blood stream through the lymphatic system and so lymph-node irradiation with IART^®^ would also be possible.

The rapid renal elimination of labelled DOTA-biotin is important for the radiation protection point of view, allowing a short period of hospitalisation (ideally one day).

IART^®^ could be an interesting nuclear medicine procedure for breast irradiation similar to the other techniques of partial breast irradiation, but offering many practical advantages over other methods.

One of the main advantages is its potential applicability to every kind of breast cancer patient scheduled for conserving surgery, without limitations of tumour location and size or multifocality. Importantly, with IART^®^, the irradiation field is identified with precision by the surgeon, who knows exactly where the tumour was located and therefore injects avidin under visual control directly into the tumour bed, thus preparing the remaining mammary gland to receive ^90^Y-biotin. With a surgeon of average experience, avidin injection around the tumour bed should be simple and uniform throughout the target area of the breast. Another IART^®^ advantage is that neither dedicated linear accelerator nor other sophisticated devices are needed. IART^®^ is a procedure that may be applied worldwide in all hospitals where breast surgery is performed and a nuclear medicine unit is present. Moreover, the possibility to inject ^90^Y-radio-labelled biotin 16–24 h after avidin administration makes the procedure suitable even if the nuclear medicine department is not close to the surgical unit. A future possible clinical scenario could be the production of ^90^Y-radio-labelled biotin in a GMP central radio pharmacy and delivered within few hours to the surrounding hospitals. This should facilitate a worldwide use of BCS and accelerated radiotherapy especially when logistical barriers to travelling are present, with consequent rebound on both the patient’s quality of life and socioeconomic aspects.

**Figure 5:**
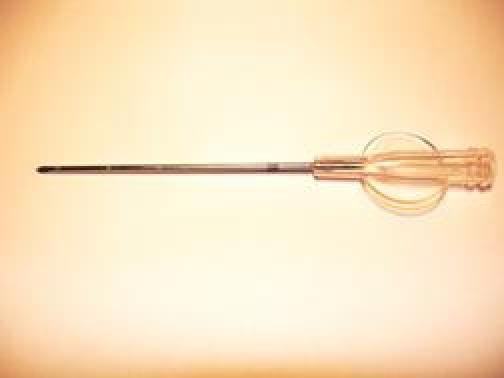

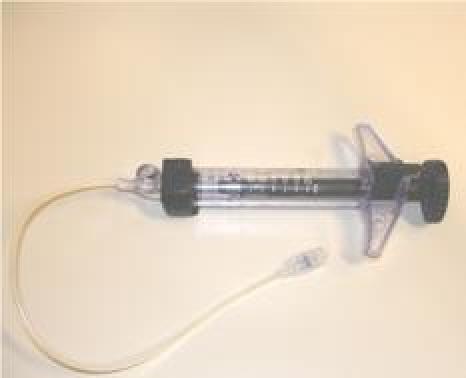
a: A multi-hole needle conceived in order to deliver avidin at different depths into the breast parenchyma at each injection site. b: A screw syringe designed to deliver an equal volume (0.7 ml) of avidin in the surgical bed.

A short learning curve would be required for the team (surgeons, radiotherapists and nuclear medicine physicians), and in our opinion, five-to-ten procedures are a suitable number to ensure a good degree of self-confidence with this approach. A possible disadvantage of this technique could be an inhomogeneous distribution of the radiopharmaceutical, as a direct consequence of the surgeon’s poor attention during the avidin injection. As a consequence, the areas of breast parenchyma, which receive more avidin, may be more irradiated, although the overnight gap between avidin and biotin injection should equilibrate the avidin tissue concentration. In order to overcome this possible negative aspect, a special multi-hole needle (Figure 5), attached to a screw syringe (Figure 5b), has been investigated by our group. These devices should ensure the administration, at different depths, of an equal defined volume of avidin into each injection site. Moreover, in order to evaluate the distribution homogeneity of ^90^Y-biotin, we also calculated the equivalent uniform dose (EUD) to the target area, applying voxel dosimetry. The EUD/BED ratio in the targeted tissue resulted to be 0.91. This value, obtained due to the crossfire effect of ^90^Y-biotin, indicates a biological efficacy comparable to that of uniform dose distribution.

Regarding the amount of radio-labelled biotin injected, we may increase the specific activity of ^90^Y-DOTA-biotin (30–40 GBq/mg), so that increasing the dose to the target breast and reducing the amount of injected activity. This is demonstrated by a previous study [25], which confirmed that high radiochemical purity (>99%) was routinely achieved with ^90^Y-DOTA-biotin prepared at the specific activities used in this study and even at tenfold higher levels (up to 40 GBq/mg). In the future, using higher specific activities of ^90^Y-biotin-DOTA, it is likely that the kidneys, bone marrow and whole body doses can be further reduced by 10%–20%. This can be aim of future investigations.

Moreover, as for IORT plus EBRT [26], adopting a treatment protocol of IART^®^ followed by 14 sessions of EBRT (spread over 2.5 weeks), a patient can save about 50% of the time required either by standard EBRT + a final boost (normally provided in 25–30 radiotherapy sessions spread over five to six weeks), or by the other accelerated radiotherapy schemes (normally spread over approximately five weeks) [27]. In addition, the scheme for IORT + EBRT is very similar to the combined IART^®^+EBRT protocol; however, a dedicated linear accelerator (or other sophisticated devices) are not required, so the procedure can be carried out wherever a nuclear medicine unit is present. This would facilitate a wider use of conservative breast surgery followed by post-operative radiotherapy.

The value of the boost has been demonstrated by studies like the ‘EORTC boost versus no-boost trial’ [28, 29], which has shown that patients who did not receive an electron boost to the tumour bed may have a worse clinical outcome than patients who received such a boost.

Moreover, an anticipated boost may entail a shortened gap between surgical removal of the tumour and the start of radiotherapy. This is considered crucial to prevent repopulation from neoplastic cellular clones present in microscopic residual disease. Indeed, after surgery, an ‘accelerated repopulation’ may occur, with an early phase in which neoplastic cells grow in an exponential way. The delivery of a boost of radiation immediately after surgery may avoid such a process and is discussed below.

Several studies support the rationale of intra-operative radiation therapy (RT) as an adjuvant therapy after surgery, including systematic reviews [30]. Specifically, a short time interval between surgery and RT should increase the probability of local tumour control [31]. An analysis of the surviving fraction (SF) and the tumour control probability (TCP), evaluated after different schemes of radiation delivery to breast cancer cells, show a decrease of the SF and an increase of the TCP when intra-operative radiation techniques are used, as compared to the standard EBRT alone.

## Conclusions

In March 2008, the study has been concluded with 38 patients enrolled. Thirty-five patients were evaluated. No side effects were observed after avidin administration and ^90^Y-biotin infusion. The dose of 100 mg of avidin resulted to be appropriate in order to deliver the radiation dose required (19.5 ± 4.0 Gy), corresponding to a BED of 21.2 ± 4.3 Gy to the surgical bed. The absorbed dose to the kidney was 3.8 ± 1.1 Gy, and no haematological toxicity was observed.

At the end of IART^®^ treatment, no local toxicity occurred and the overall cosmetic result was good. Thirty-two patients completed EBRT. The tolerance to EBRT was good with no significant difference from conventional treatment. IART^®^ was well accepted by all patients; it did not involve any changes in their quality of life. Based on these preliminary data, it seems that the entire IART^®^-EBRT procedure is generally well tolerated, with few cases of temporary local toxicity.

IART^®^ can be considered as an ‘*anticipated boost’* three to four weeks before WB irradiation with EBRT. This will turn out in a new approach for accelerated WB irradiation after BCS, with considerable economical and social impact. As for sentinel node lympho-scintigraphy and biopsy [32, 33], we hope that this nuclear medicine technique will contribute to a better management of breast cancer patients.

## Figures and Tables

**Table 1:**
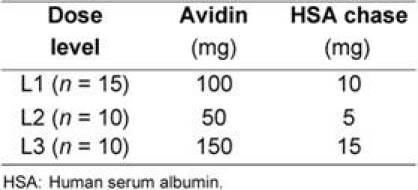
Three consecutive cohorts of patients treated with different levels of avidin. All patients received ^90^Y-ST2210 (3.7 GBq) spiked with ^111^In (185 MBq)

**Table 2:**
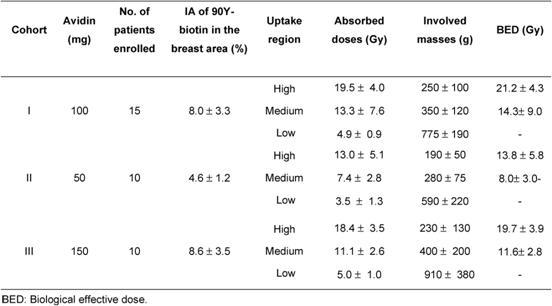
Dosimetric data (mean values ± SD) of the high-, medium- and low-uptake areas of the irradiated breast

**Table 3:**
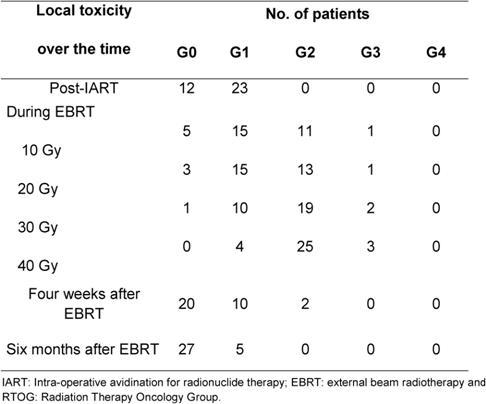
Local toxicity in 35 patients at different time points (post-IART, during EBRT, one month and six months after EBRT) evaluated by RTOG Scale

**Table 4:**
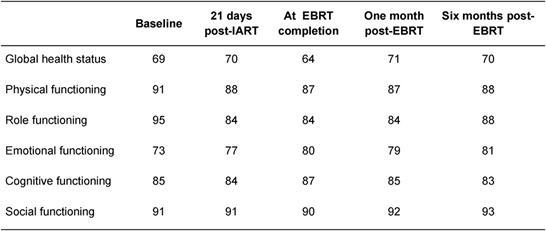
Average quality-of-life score (EORTC QOL-30 questionnaire) at various times during treatment and follow-up for global functions. A low score (minimum 0) indicates poor quality of life and a high score (maximum 100) indicates good quality of life

**Table 5:**
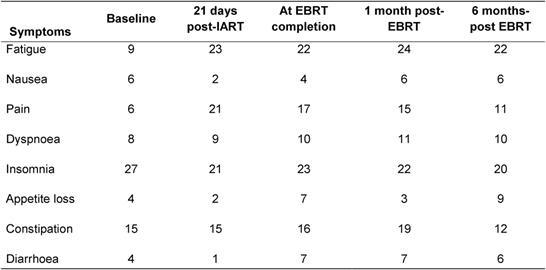
Average quality-of-life score (EORTC QOL-30 questionnaire) at various times during treatment and follow-up for symptoms. A low score (minimum 0) indicates good quality of life and a high score (maximum 100) indicates poor quality of life
